# Straw return was more beneficial to improving saline soil quality and crop productivity than biochar in the short term

**DOI:** 10.3389/fpls.2024.1517917

**Published:** 2025-01-20

**Authors:** Ping Cong, Jiashen Song, Jianxin Dong, Wenyan Su, Wenhao Feng, Hongyuan Zhang

**Affiliations:** ^1^ State Key Laboratory of Efficient Utilization of Arid and Semi-arid Arable Land in Northern China (The Institute of Agricultural Resources and Regional Planning, Chinese Academy of Agricultural Sciences, Beijing, China; ^2^ Tobacco Research Institute, Chinese Academy of Agricultural Sciences, Qingdao, China; ^3^ Graduate School of Chinese Academy of Agricultural Sciences, Beijing, China

**Keywords:** saline soil, soil quality index, crop yield, ecosystem multi-functionality, straw return, biochar

## Abstract

Salinized soil often exhibits high salt content and low nutrient availability, leading to the reduction of soil ecosystem function and crop productivity. Although straw return has profound effects on saline soil improvement, how soil quality index (SQI), soil ecosystem multifunctionality (EMF), and crop yield respond to different organic ameliorants remain unclear. Herein, a field experiment was established to explore the influence of various straw management strategies (no organic ameliorant, CK; corn straw return, CS; and corn straw biochar return; CB) on the saline soil functions and crop productivity. In relation to CK and CB, CS significantly improved SQI by 52% and 35%, respectively. This may be due to the decreased soil salt (especially soluble Na^+^) and increased available nutrients under corn straw return. Furthermore, CS increased soil EMF than CK by 71% and CB by 39%, which was caused by the increased activities of 1,4-β-glucosidase, β-1,4-*N*-acetyl-glucosaminidase, and leucine aminopeptidase. The linear model further supported that soil enzyme activities are positively related to available nutrient contents and negatively correlated with salt content. Moreover, the crop yield under CS significantly increased by 22% compared to CK. Also, soil quality positively influenced crop yield, with soil salt and available phosphorus being the primary influencing factors. However, crop yield was not sensitive to soil EMF. In summary, straw return was more beneficial to improving soil quality and crop productivity than biochar in the short term in saline soils.

## 1 Introduction

It is anticipated that the worldwide population will reach approximately 9.8 billion by 2050; the currently arable land, however, cannot meet the food requirements of the increasing population (FAO, 2018). It is therefore crucial to enhance crop sustainability and productivity by rehabilitating degraded land resources. Salinization-induced soil degradation is a major environmental problem that critically influences global agricultural productivity and sustainable development (Qadir et al., 2008; Sahab et al., 2021; Song et al., 2023). Currently, it is estimated approximately 1 billion ha of land worldwide is experiencing varying degrees of salinization, which constitutes approximately 10% of the total arable land (Wang et al., 2017). Furthermore, due to global climate change and poor irrigation as well as tillage management strategies, the area of land impacted by salt content is increasing annually by 1.5–2.5 × 10^5^ ha (Mustafa et al., 2019). Therefore, a valid, low-cost, and environmentally friendly strategy is required for the improvement of saline soils to fulfill the development of sustainable agriculture (Kheir et al., 2019; Meena et al., 2016; Song et al., 2024).

Currently, straw return to the field is widely supported as an eco-friendly method for soil improvement (Turmel et al., 2015). Numerous studies suggested that straw return could enhance saline soil health via a series of pathways such as improvement of soil aeration conditions and structure, promotion of salt leaching and nutrient recycling, and provision of energy for microorganisms (Urraa et al., 2018; Xie et al., 2020; Ibrahim et al., 2020; Zhang et al., 2020; Song et al., 2023). Nevertheless, straw return generally induces a positive priming effect on native soil organic carbon (SOC) mineralization by enhancing unstable organic C and particular soil microorganisms (Xu et al., 2019). Furthermore, there has been a rising focus on the effects of biochar application on saline soil improvement in recent years. Biochar is produced through the pyrolytic carbonization of organic substances (i.e., straw) under high temperatures and anaerobic environments (Lehmann and Joseph, 2015; Wu et al., 2024). Because of its preferable stability, porosity, and extensive specific surface area, straw biochar has proven significant effectiveness in decreasing soil salt content, improving soil microstructure, reducing nutrient leaching and enhancing soil fertility, and boosting microbial and enzyme activities in saline soil (Mahmoud et al., 2019; Akhtar et al., 2015; Aborisade et al., 2023). However, straw biochar may also promote the oxidation of volatile substances and surface functional groups (Singh et al., 2010). Once passivated, corn straw biochar interacts with soil, creating a protective matrix (Singh et al., 2010). Although many studies have found the benefits of the application of straw and biochar on individual soil index and function, there is a limited number of comprehensive frameworks designed to assess soil quality that integrate various indexes into a synthetic index (Gunasekaran et al., 2021; Paz-Ferreiro et al., 2017).

Recently, there has been an incremental emphasis on soil ecosystem multifunctionality (EMF) for assessing the intricate interactions among biological, geochemical, and physical processes (Wittwer et al., 2021). Soil extracellular enzymes are proteins exhibiting notable catalytic activity that are released by crop roots and microorganisms (Zhou et al., 2023). These enzymes are associated with shifts in soil microbial characteristics and are able to indicate the status of soil nutrients. Consequently, they are frequently employed as key indicators for assessing soil ecosystem functions (Xue et al., 2020; Song et al., 2024). With the addition of different organic ameliorants, the changes in soil salt content and fertility can greatly influence microbial metabolism, thus affecting the utilization and assimilation of nutrients by microorganisms via the production of extracellular enzymes, and ultimately can exert various effects on soil EMF (Stark et al., 2014; Zhang et al., 2020). However, how different organic ameliorants affect soil enzyme activities and EMF by regulating soil physicochemical properties reflected in soil quality is not clear. Notably, straw and biochar additions can supply the soil with a substantial amount of exogenous nutrients, alleviate the constraints on microbial nutrient utilization, and enable crops to efficiently absorb and utilize nutrients for high yields (Singh et al., 2016). Nevertheless, due to the high C/N ratio of these organic materials, their applications also reduce crop yield by encouraging soil microorganisms to secrete extracellular enzymes, which compete with crops for available nutrients (Xiao et al., 2022). Therefore, a thorough understanding of the changes in soil quality and EMF, along with their relationship with crop yield, is essential for establishing compatible management strategies to rehabilitate saline soils.

In order to fulfill these knowledge gaps, a field experiment was performed in 2023 to assess the variations of soil quality, EMF, as well as crop yield under different organic ameliorants (no organic ameliorant, corn straw return, and corn straw biochar return) in saline soil. We aimed to i) identify which straw return strategy is better to improve soil quality, soil EMF, and crop productivity in saline soils in the short term and ii) determine the relationship among soil quality, soil EMF, and crop yield under different organic ameliorants in saline soils. We hypothesized that i) organic ameliorants could enhance soil quality by decreasing soil salt, increasing soil nutrient contents, and further enhancing soil EMF; ii) however, crop yield may be more sensitive to saline soil quality than soil EMF; and iii) compared to straw biochar, straw return may be better at improving saline soil quality and crop productivity in the short term.

## 2 Materials and methods

### 2.1 Study site

The field experiment was carried out in Nonggao District (37°02′N, 118°25′E), Guangrao County, Shandong, China. This region exhibits a warm temperate continental monsoon climate. The average annual precipitation and temperature are 532 mm and 12.3°C, respectively. Meteorological data in 2023 are presented in **Supplementary Figure S1**. The soil at the experimental site is a typical coastal saline soil, and the soil properties at 0–20 cm, before the experiment started, were as follows: pH value of 8.46, a salt content of 1.88 g kg^−1^; soluble K^+^, Ca^2+^, Na^+^, Mg^2+^, Cl^−^, SO_4_^2−^, and HCO_3_^−^ of 0.03, 0.13, 1.52, 0.10, 0.05, 0.45, and 0.03 g kg^−1^, respectively; SOC of 7.6 g kg^−1^; total nitrogen (N) of 1.01 g kg^−1^; and available N, phosphorus (P), and potassium (K) of 50.41, 34.98, and 393.71 mg kg^−1^, respectively.

### 2.2 Experimental design

The study area is an abandoned land without tillage and fertilization before 2023. The study was established in 2023 and included three treatments with random design: i) CK, no organic ameliorant; ii) CS, corn straw return; and iii) CB, corn straw biochar return. Each treatment was conducted in triplicate, with individual plots measuring 30 m^2^ (3 m × 10 m). Before starting the experiment, corn straw (15 t ha^−1^) and corn straw biochar (8 t ha^−1^) were thoroughly mixed into the 0–10-cm soil through plowing. The corn straw returning amount was based on the high straw yield (15 t ha^−1^) in the local region. Following the principle of equal C input and referring to the C content in straw and biochar (shown in **Supplementary Table S1**), the application rate of biochar was determined to be 8 t ha^−1^ (Wang et al., 2024). Among them, corn straw was obtained from local corn fields, dried, and crushed before application. Corn straw biochar was prepared from the abovementioned corn stover in an anaerobic environment at 700°C (Cong et al., 2022). The nutrient content of corn straw and straw biochar is presented in **Supplementary Table S1**. The spring corn variety was Ludan 506, sown on May 10, 2023, with a row spacing of 0.7 m for wide rows, 0.5 cm for narrow rows, plant spacing, and planting density of 0.2 m, and 90,000 plants ha^−1^. Additionally, 750 kg ha^−1^ of controlled-release fertilizer was applied with an NPK ratio of 28:6:6, and 300 kg ha^−1^ of urea was added during the large mouth period. After sowing and during the big mouth period, water was irrigated twice with a volume of 750 m^3^ ha^−1^ each time. Other management strategies followed the standard local conventional planting methods.

### 2.3 Soil and crop sampling and analysis

After harvesting in 2023, 0–20-cm soil samples were obtained using a soil drill with a three-point sampling method. After removing roots, soil samples were divided into two portions. One subsample was kept at room temperature for analysis of soil salt; soluble ions; total N; available N, P, and K; and SOC; all of them were used to calculate the soil quality index (SQI) (Lu, 2000; Bao, 2010). The second subsample was stored at 4°C in a refrigerator for up to 2 weeks to measure soil enzyme activities, including β-glucosidase (BG), cellobiosidase (CE), β-*N*-acetyl-glucosaminidase (NAG), and leucine aminopeptidase (LAP), which were used to calculate the soil EMF (Marx et al., 2001). Detailed methods for measuring soil properties are provided in **Supplementary Table S2**. The corn grain samples (two rows, 5 m long) were collected in each plot. After air-drying, the samples were threshed to precisely assess the corn yield.

### 2.4 Calculations

#### 2.4.1 SQI

To assess SQI, each soil property was initially transformed into a value (0–1) through the appropriate equation listed below. The soil properties were then grouped into two categories. If a given soil property improved with soil quality (total N; available N, P, and K; and SOC), the “more is better” approach was used, applying Equation 1. For a soil property where lower values signify better quality (salt content), the “less is better” method was used with Equation 2 (Kuzyakov et al., 2020; Zhou et al., 2020; Song et al., 2024):


(1) $$Li=\frac{v}{v_{max}}$$

(2) $$Li=\frac{v_{min}}{v}$$

where *Li* refers to the linear score of soil property *i*, and *v*, *v_max_*, and *v_min_* refer to the measured, maximum, and minimum values of the soil property *i*, respectively.

SQI was then determined using the SQI-area method. It involves evaluating the area encompassed through the radar diagram created from all soil properties (Kuzyakov et al., 2020):


(3) $$SQI=0.5\times \sum_{i}^{n}Li^{2}\times \text{sin}\left(\frac{2\times \pi }{n}\right)$$

where *n* represents the total number of soil properties.

#### 2.4.2 Soil EMF

Enzyme activities were utilized to evaluate soil ecosystem multifunctionality (Delgado-Baquerizo et al., 2020). The Z-score approach was applied to normalize each soil enzyme activity, after which the average values were calculated (Delgado-Baquerizo et al., 2016):


(4) $$Z_{i}=\frac{x-m_{i}}{sd}$$

(5) $$Z_{Score}=average\left(Z_{i}\right)$$

where *Z_i_* refers to enzyme activity, and *x*, *m_i_*, and *s_d_* correspond to the measured enzyme activity, mean enzyme activity, and its standard deviation, respectively.

### 2.5 Statistical analysis

One-way analysis of variance (ANOVA) was carried out to explore the effect of organic ameliorants (CK, CS, and CB) on soil physicochemical properties, enzyme activities, and corn yield. Multiple comparisons were conducted using Fisher’s least significant difference (LSD) test, with a significance threshold of *p* < 0.05. The relationships among SQI, soil EMF, and crop yield were investigated using a linear regression model. Random Forest analysis was carried out to confirm the important factors of crop yield among soil physicochemical properties using R (“randomForest” package). All figures were drawn using OriginPro 2021, and statistical analyses were carried out using DPS 9.01.

## 3 Results

### 3.1 Soil physicochemical properties and SQI

**Figure 1:**
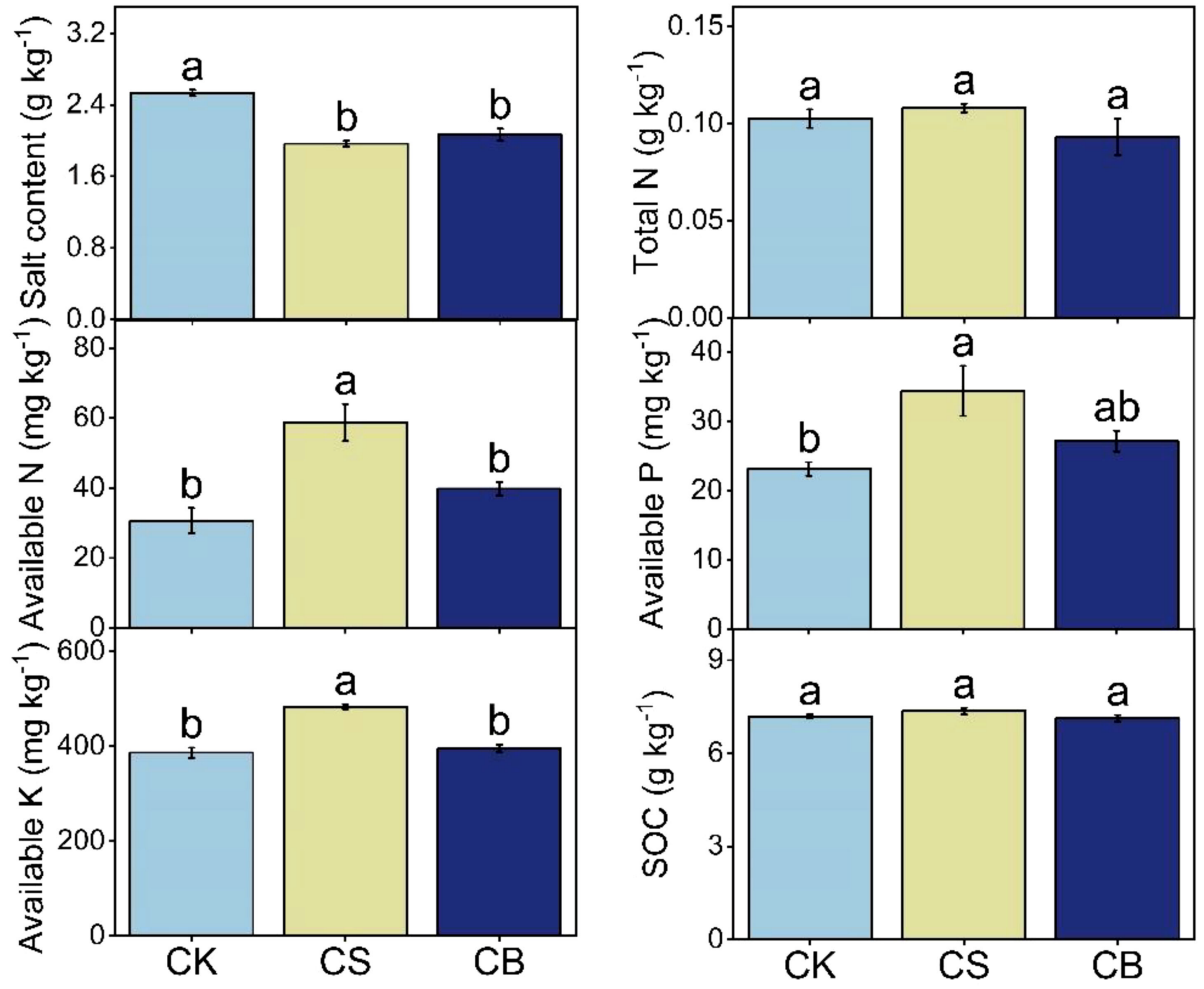
Soil physicochemical properties at 0–20 cm as affected by organic ameliorants. Organic ameliorants were as follows: CK, no organic ameliorant; CS, corn straw return; and CB, corn straw biochar return. N, nitrogen; P, phosphorus; K, potassium; SOC, soil organic carbon. Bars were SE, and letters were least significant difference (LSD) at *p* < 0.05 (n = 3).

**Figure 2:**
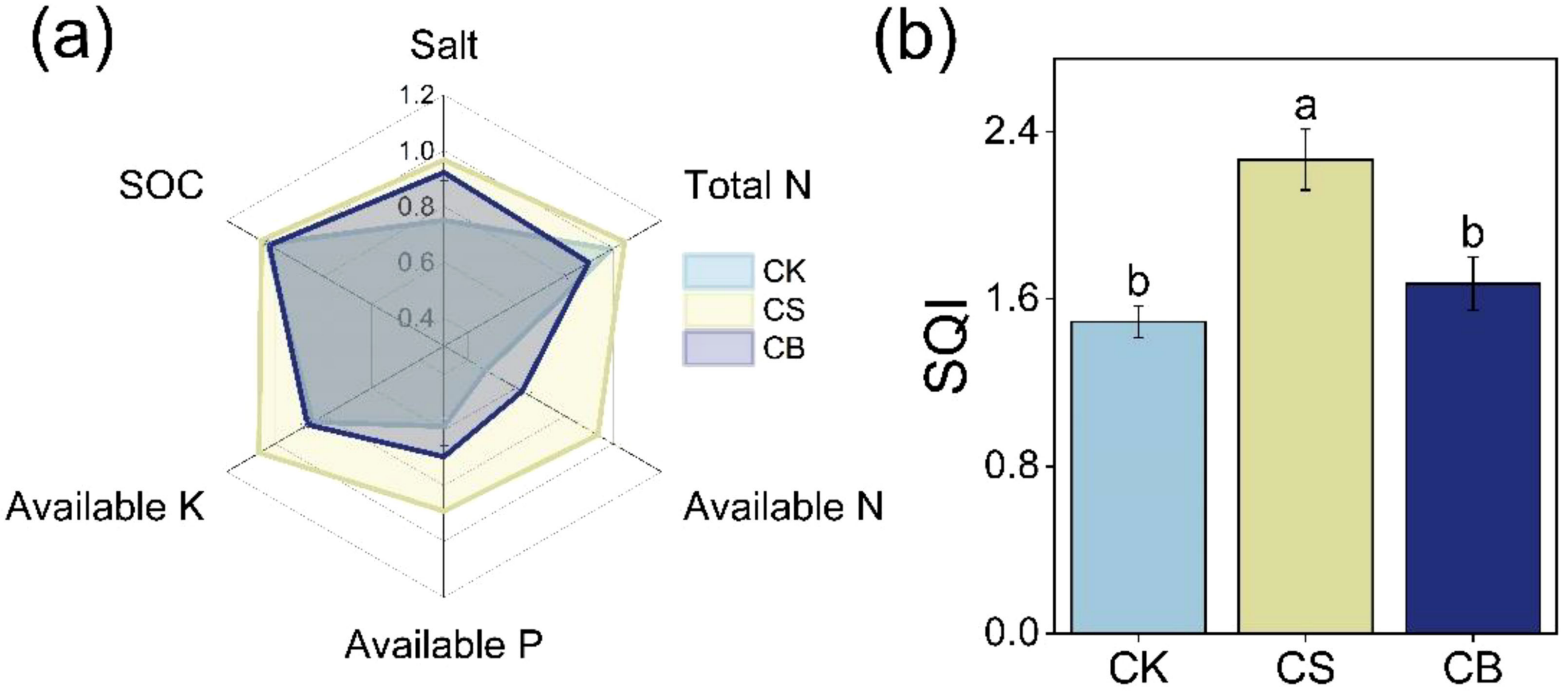
Soil physicochemical property scores at the 0–20 cm under organic ameliorants **(A)** and soil quality index (SQI) under organic ameliorants **(B)**. Organic ameliorants were as follows: CK, no organic ameliorant; CS, corn straw return; CB, corn straw biochar return. N, nitrogen; P, phosphorus; K, potassium; SOC, soil organic carbon. Bars are SE, and letters are least significant difference (LSD) at *p* < 0.05 (n = 3).

CS and CB decreased soil salt content by 22% and 18%, respectively; CS increased soil available N, available P, and available K by 91%, 49%, and 25% as compared to CK, respectively (*p* < 0.05, **Figure 1**). CS then improved SQI by 52% as compared to CK (*p* < 0.05, **Figure 2**). Furthermore, the SQI was 35% higher in CS than in CB by 35%.

### 3.2 Soil enzyme activity

**Figure 3:**
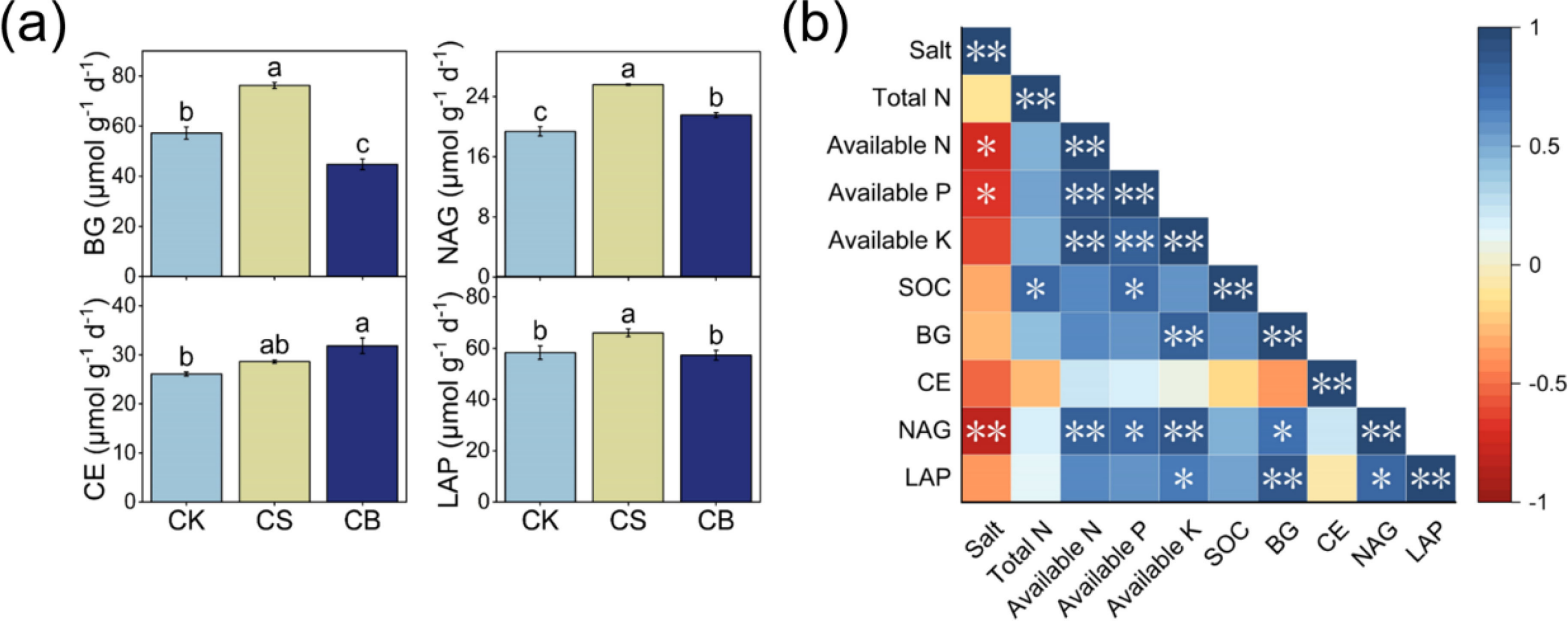
Soil enzyme activity at 0–20 cm under organic ameliorants **(A)**. Pairwise comparisons between soil properties at 0–20 cm **(B)**. Organic ameliorants were as follows: CK, no organic ameliorant; CS, corn straw return; CB, corn straw biochar return. BG, 1,4-β-glucosidase; CE, cellobiosidase; NAG, β-1,4-*N*-acetyl-glucosaminidase; LAP, leucine aminopeptidase. Bars were SE, and letters were least significant difference (LSD) at *p* < 0.05 (n = 3). *p < 0.05, **p < 0.01.

Soil enzyme activity responded differently to various organic ameliorants (**Figure 3A**). CS significantly increased the activities of BG, NAG, and LAP by 33%, 32%, and 13%, respectively; CB significantly increased the activities of CE and NAG by 22% and 11% as compared to CK, respectively (*p* < 0.05, **Figure 2A**). Furthermore, compared to CB, CS increased BG, NAG, and LAP activities by 70%, 19%, and 15%, respectively (*p* < 0.05, **Figure 3A**).

Pairwise comparisons suggested that there are negative correlations between NAG activity and salt content (*p* < 0.05, **Figure 3B**); positive correlations between the activities of BG, NAG, LAP, and available K; and positive correlations between the NAG activity and available N and P (*p* < 0.05).

### 3.3 Soil ecosystem multifunctionality

**Figure 4:**
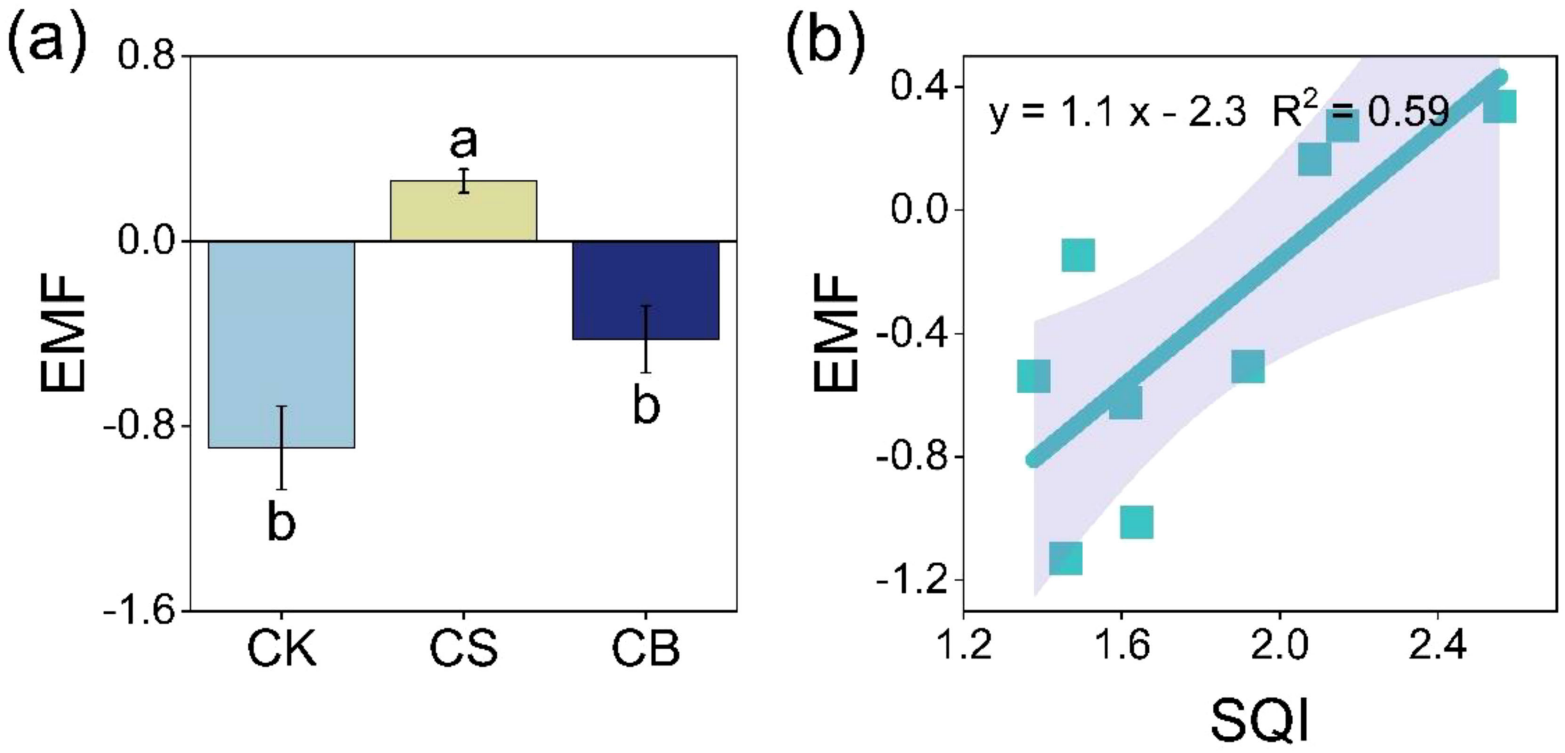
Soil ecosystem multifunctionality (EMF) **(A)** and its relationship with soil quality index (SQI) **(B)** at 0–20 cm as affected by organic ameliorants. Organic ameliorants were as follows: CK, no organic ameliorant; CS, corn straw return; CB, corn straw biochar return. Bars were SE, and letters were least significant difference (LSD) at *p* < 0.05 (n = 3).

CS increased soil EMF by 71% and 39% as compared to CK and CB, respectively (*p* < 0.05, **Figure 4A**). The linear regression analysis indicated a positive correlation between soil EMF and SQI (*p* < 0.05, **Figure 4B**).

### 3.4 Crop yield and its driving factors

**Figure 5:**
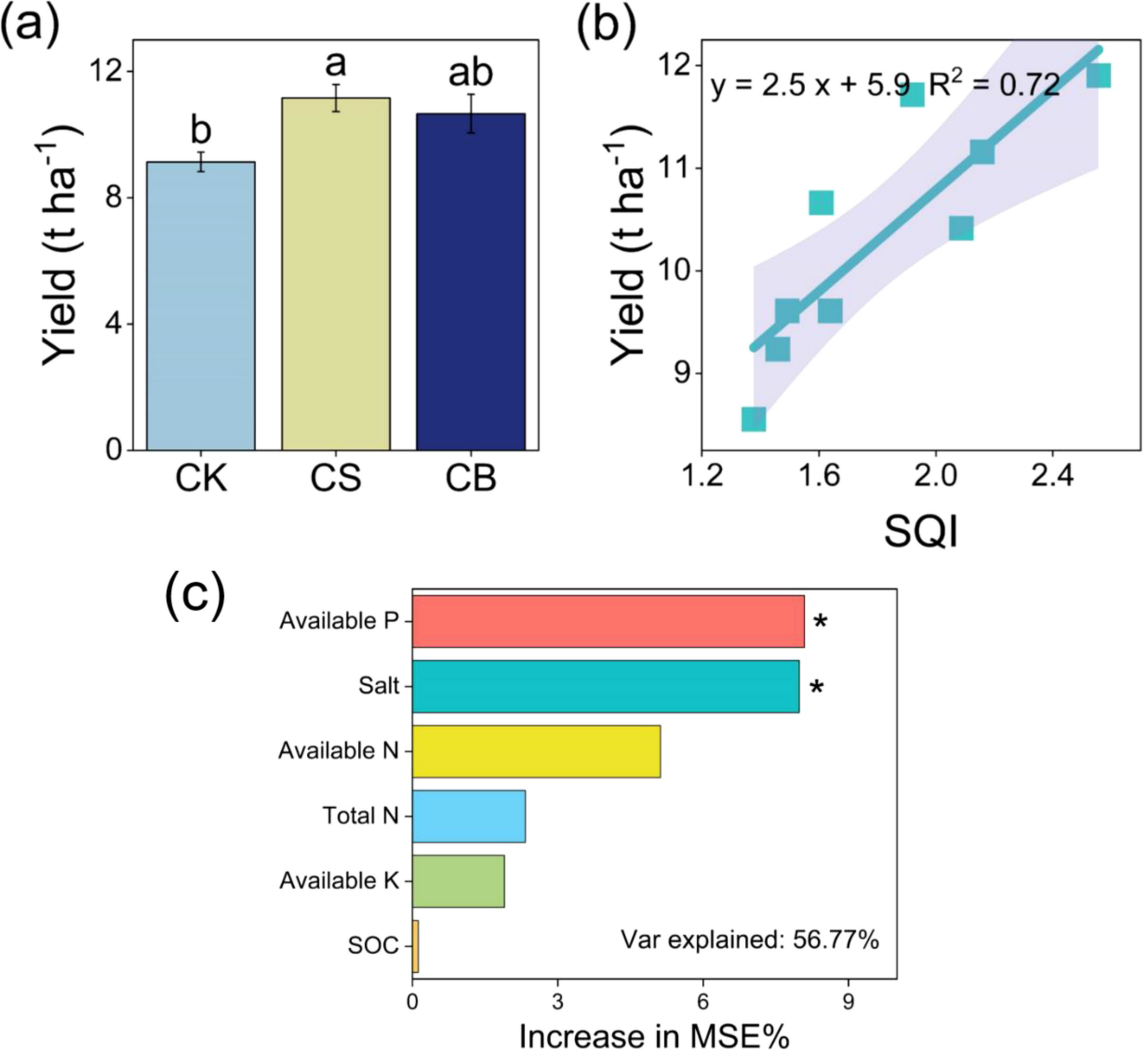
Crop yield **(A)** and its relationship with soil quality index (SQI) **(B)** at 0–20-cm depth as affected by organic ameliorants. Random Forest indicates predictor importance (% of increase of MSE) of soil physicochemical properties on crop yield **(C)**. Organic ameliorants were as follows: CK, no organic ameliorant; CS, corn straw return; CB, corn straw biochar return. MSE, mean squared error. Bars are SE, and letters are least significant difference (LSD) at p < 0.05(n = 3). *p < 0.05.

Compared with CK, crop yield increased by 22% under CS (*p* < 0.05, **Figure 5A**). Nevertheless, there was no difference in crop yield between CB and CS (*p* > 0.05). The linear regression indicated that crop yield was positively correlated with SQI (*p* < 0.05), while the relationship between crop yield and soil EMF was not significant (*p* > 0.05, **Figure 5B**). The dominating factors of soil physicochemical properties related to crop yield were available P and salt content (**Figure 5C**).

## 4 Discussion

### 4.1 Effects of organic ameliorants on soil quality

Soil quality refers to the ability of soil to operate efficiently within ecosystem limits, supporting biological productivity, preserving environmental quality, and supporting the health of plants, animals, and humans (Doran and Parkin, 1994; Liu et al., 2023). Our results suggested that saline soil quality considerably increased under corn straw return (**Figure 2**). This may be due to the decreased soil salt and increased available nutrient contents (**Figure 1**). In the decomposition process of corn straw, organic acids were released, which subsequently displaced Na^+^ on soil colloids, ultimately decreasing salt content (Fan et al., 2013). This was also supported by the decreased soil soluble Na^+^ content under straw return treatment (**Supplementary Figure S2**). Furthermore, applying corn straw could enhance soil aggregation and progressively rehabilitate soil structure (Zhang et al., 2020), thereby promoting soil salt leaching (Benbi and Senapati, 2010; Abdelrhman et al., 2021). The improvement of soil saline environment and physical structure, on the one hand, increased soil nutrient (i.e., N, P, and K) availability by increasing crop root biomass and, on the other hand, created a suitable niche for microbial growth and subsequently enhanced enzyme activity to activate nutrient transformation and immobilization (Song et al., 2018; Wang et al., 2021). Moreover, the decomposition of corn straw produced organic matter, such as humus, which possessed an extensive surface area and significant adsorption capability (Chen et al., 2020). This, in turn, led to a decrease in nutrient leaching; consequently (Xu et al., 2020), an increase in soil nutrient contents was observed under straw return. Although the organic matter contained in the corn straw was continuously decomposed and released, which increased SOC and available nutrient contents (Tian et al., 2020), corn straw return could also contribute to a positive priming effect on the mineralization of persistent SOC (Xu et al., 2019). Therefore, SOC remained stable when straw was returned to the soils in our case.

The application of straw biochar, with its porous structure, large surface area, and strong hydroxyl group adsorption capacity, could enhance the leaching of both salts and sodium (Lakhdar et al., 2009) and, as a consequence, decreased soil salt content under straw biochar return (**Figure 1**). The reduced soil soluble Na^+^ content under straw biochar return treatment also supported this point (**Supplementary Figure S2**). Nevertheless, the changes in available nutrients and SOC contents were not significant after biochar addition (**Figure 1**). This may be due to the addition of corn straw biochar that stimulated the oxidation of volatile substances and surface functional groups (Singh et al., 2010). Subsequently, the passivated corn straw biochar interacted with the soil, forming a protective matrix (Singh et al., 2010). The C contained in corn straw biochar could also alter the abundance, composition, and activities of specific microorganisms, leading to the mineralization of native SOC. Afterward, there was no remarkable variation in SOC content under corn straw biochar return. Overall, corn straw return could quickly improve soil quality by decreasing soil salt and improving available nutrient contents, while biochar application could not improve soil quality in saline soils in the short term.

Here, it should be noted that the higher availability of mineral elements (available N and P) with the application of organic ameliorants may lead to environmental pollution such as nitrate leaching and N_2_O emissions. Further studies are required to evaluate the effect of organic ameliorants on greenhouse gas emissions and nitrate leaching.

### 4.2 Effects of organic ameliorants on soil EMF

Understanding soil functions is essential for assessing the ecological benefits of various agricultural management strategies (Wittwer et al., 2021). In our study, corn straw return enhanced soil EMF compared with CK (**Figure 3A**), which indicated that corn straw return may mitigate some of the adverse impacts associated with chemical fertilization (Wittwer et al., 2021). This result could be attributed to the application of labile C sources under corn straw return, which enhanced the activities of enzymes involved in C and N acquisition (Zhou et al., 2024). This, in turn, stimulated the secretion of enzymes by microbes and ultimately led to the increase of soil EMF (Jia et al., 2022). Additionally, enzyme activities were significantly influenced by environmental factors such as soil salt content and nutrient availability (Liang et al., 2005). The increase in microbial activity caused by decreasing salt content and improving nutrient availability under organic ameliorants also led to the enhancement of enzyme activity of nutrient transformation to further increase nutrient immobilization (Song et al., 2018; Wang et al., 2021). The linear model showing that enzyme activities were positively related to available nutrient contents and negatively related to salt content supported these points (**Figure 3B**). Therefore, the improvement of soil salt and available nutrient contents under corn straw return could greatly influence microbial metabolism (Zhang et al., 2020), thus promoting the utilization and assimilation of nutrients by microorganisms through the production of enzymes (**Figure 2**), and then had a positive effect on soil EMF (**Figure 3A**; Zhang et al., 2020). This was also confirmed by a significant positive correlation between soil quality and EMF (**Figure 3B**). Nevertheless, there was no remarkable variation in soil EMF under corn straw biochar return (**Figure 3A**). The potential explanation was that corn straw biochar had the ability to reduce microbial growth and turnover by fixing soil nutrients, which in turn inhibited the increase of enzyme activities and soil EMF (Kalu et al., 2024).

### 4.3 Effects of organic ameliorants on crop yield

The primary objective of improving saline soil is to elevate soil quality and, more importantly, to attain a higher crop yield within a shorter term (Shrivastava and Kumar, 2015). In our study, corn straw return remarkably improved crop yield (**Figure 5A**). This may be because the addition of straw improved soil quality and facilitated the efficient absorption and utilization of nutrients by crops, thus increasing crop yield (Song et al., 2022). It was further confirmed by the significant positive correlation between SQI and crop yield (**Figure 5B**). The Random Forest result further suggested that soil salt and available P were the main factors influencing crop yield (**Figure 5C**). First, higher salt content previously could disrupt the dynamic water balance in the crop, which in turn affects their nutrient balance. After the straw return, the improvement of soil saline environment and physical structure also increased root development and further crop yield by increasing soil nutrients (i.e., N, P, and K) (Zhao et al., 2020). Second, the straw-induced enhanced P may overwhelm the severe P deficiency caused by strong adsorption between soil particles and P elements in saline soils (Li and Li, 2022). Unlike soil quality, crop yield was not affected by soil EMF (**Supplementary Figure S3**). This suggested that an increase in soil enzyme activity may not necessarily be advantageous for crop growth because of the high C/N ratio in biochar, which may intensify the competition between soil microorganisms and crops for available nutrients through the secretion of enzymes, thereby potentially negatively impacting crop growth and yield under biochar addition (Xiao et al., 2022). Therefore, compared to corn straw biochar return, corn straw return was more likely to improve saline soil quality in the short term, thereby enhancing EMF and crop yield, and was more conducive to rapid improvement of saline soils.

## 5 Conclusion

Corn straw return reduced soil salt, increased available nutrient contents, and then increased soil quality than CK and corn straw biochar return. Furthermore, soil salt (mainly soluble Na^+^) and available nutrient contents were negatively correlated and positively correlated with enzyme activities, respectively. Therefore, enzyme activities and soil EMF increased under corn straw return than that under CK and corn straw biochar return. Moreover, higher soil quality also led to higher crop yield. Compared to CK and straw biochar return, straw return significantly increased crop yield. The Random Forest result suggested that soil salt and available phosphorus contents were the main driving factors for improving soil quality. In conclusion, straw return was more beneficial for improving soil quality and ecosystem multifunctionality, as well as crop yield, and providing references for rapid improvement of saline soils.

## Data Availability

The raw data supporting the conclusions of this article will be made available by the authors, without undue reservation.

## References

[B1] Abdelrhman A. A. Gao L. Li S. P. Lu J. J. Song X. J. Zhang M. N.. (2021). Long-term application of organic wastes improves soil carbon and structural properties in dryland affected by coal mining activity. Sustainability 13, 5686. doi: 10.3390/su13105686

[B2] Aborisade M. A. Feng A. Oba B. T. Kumar A. Battamo A. Y. Huang M.. (2023). Pyrolytic synthesis and performance efficacy comparison of biochar-supported nanoscale zero-valent iron on soil polluted with toxic metals. Arch. Agron. Soil Sci. 69, 2249–2266. doi: 10.1080/03650340.2022.2146100

[B3] Akhtar S. S. Andersen M. N. Liu F. (2015). Residual effects of biochar on improving growth, physiology and yield of wheat under salt stress. Agric. Water Manage. 158, 61–68. doi: 10.1016/j.agwat.2015.04.010

[B4] Bao S. D. (2010). Soil agrochemical analysis (Beijing: China Agricultural Press), 495.

[B5] Benbi D. K. Senapati N. (2010). Soil aggregation and carbon and nitrogen stabilization in relation to residue and manure application in rice–wheat systems in Northwest India. Nutrient Cycling Agroecosystems 87, 233–247. doi: 10.1007/s10705-009-9331-2

[B6] Chen X. D. Wu J. G. Opoku-Kwanowaa Y. (2020). Effects of returning granular corn straw on soil humus composition and humic acid structure characteristics in saline alkali soil. Sustainability 12, 1005. doi: 10.3390/su12031005

[B7] Cong P. Song S. H. Song W. J. Dong J. X. Zheng X. B. (2022). Biochars prepared from biogas residues: temperature is a crucial factor that determines their physicochemical properties. Biomass Conversion Biorefnery 14, 12843–12856. doi: 10.1007/s13399-022-03229-y

[B8] Delgado-Baquerizo M. Grinyer J. Reich P. B. Singh B. K. (2016). Relative importance of soil properties and microbial community for soil functionality: insights from a microbial swap experiment. Funct. Ecol. 30, 1862–1873. doi: 10.1111/fec.2016.30.issue-11

[B9] Delgado-Baquerizo M. Reich P. B. Trivedi C. Eldridge D. J. Abades S. Alfaro F. D.. (2020). Multiple elements of soil biodiversity drive ecosystem functions across biomes. Nat. Ecol. Evol. 4, 210–220. doi: 10.1038/s41559-019-1084-y 32015427

[B10] Doran J. W. Parkin T. B. (1994). “Defining and assessing soil quality,” in Defining soil quality for a sustainable environment. Eds. Doran J. W. Coleman D. C. Bezdicek D. F. (Madison,WI: Soil Science Society of America), 3–21. Book Series No. 35.

[B11] Fan F. Zhang Q. G. Hou F. H. Tai J. C. Sun D. Z. Liu Z. W. (2013). Effects of corn straw isolation layer on alkalization characteristics and nutrient status of saline-alkali soil in the West Liaohe River Basin. J. Soil Water Conserv. 27, 131–137. doi: 10.13870/j.cnki.stbcxb.2013.03.029

[B12] FAO. (2018). Global Livestock Environmental Assessment Model: Model Description Version 2.0. Food and Agricultural Organisation of the United Nations (FAO) report. Available online at: https://www.fao.org/fileadmin/user_upload/gleam/docs/GLEAM_2.0_Model_description.pdf.

[B13] Gunasekaran Y. Kaliappan S. B. Porpavai S. (2021). Developing soil quality indices for different crop rotations of deltaic inceptisol regions of India. Commun. Soil Sci. Plant Anal. 52, 1363–1376. doi: 10.1080/00103624.2021.1885683

[B14] Ibrahim M. Mahmoud E. Ibrahim D. (2020). Assessing the impact of water treatment residuals and rice straw compost on soil physical properties and wheat yield in saline sodic Soil. Commun. Soil Sci. Plant Anal. 51, 2388–2397. doi: 10.1080/00103624.2020.1836206

[B15] Jia R. Zhou J. Chu J. Shahbaz M. Yang Y. D. Jones D. L.. (2022). Insights into the associations between soil quality and ecosystem multifunctionality driven by fertilization management: A case study from the North China Plain. J. Cleaner Production 362, 132265. doi: 10.1016/j.jclepro.2022.132265

[B16] Kalu S. Seppänen A. Mganga K. Z. Sietiö O. M. Glaser B. Karhu K. (2024). Biochar reduced the mineralization of native and added soil organic carbon: evidence of negative priming and enhanced microbial carbon use efficiency. Biochar 6, 7. doi: 10.1007/s42773-023-00294-y

[B17] Kheir A. M. S. Abouelsoud H. M. Hafez E. M. Ali O. A. M. (2019). Integrated effect of nano-Zn, nano-Si, and drainage using crop straw–filled ditches on saline sodic soil properties and rice productivity. Arabian J. Geosciences 12, 471. doi: 10.1007/s12517-019-4653-0

[B18] Kuzyakov Y. Gunina A. Zamanian K. Tian J. Luo Y. Xu X. (2020). New approaches for evaluation of soil health, sensitivity and resistance to degradation. Front. Agric. Sci. Eng. 7, 282–228. doi: 10.15302/J-FASE-2020338

[B19] Lakhdar A. Rabhi M. Ghnaya T. Montemurro F. Jedidi N. Abdelly C. (2009). The effectiveness of compost uses in the salt-affected soil. J. Hazardous Materials 171, 29–37. doi: 10.1016/j.jhazmat.2009.05.132 19576686

[B20] Lehmann J. Joseph S. (2015). Biochar for environmental management: science, technology and implementation. 2nd ed (Abingdon, UK: Routledge).

[B21] Li Y. F. Li G. H. (2022). Mechanisms of straw biochar’ s improvement of phosphorus bioavailability in soda saline−alkali soil. Environ. Sci. pollut. Res. 29, 47867–47872. doi: 10.1007/s11356-022-20489-3 35522415

[B22] Liang Y. Si J. Nikolic M. Peng Y. Chen W. Jiang Y. (2005). Organic manure stimulates biological activity and barley growth in soil subject to secondary salinization. Soil Biol. Biochem. 37, 1185–1195. doi: 10.1016/j.soilbio.2004.11.017

[B23] Liu C. Feng X. Xu Y. Kumar A. Yan Z. Zhou J.. (2023). Legume-based rotation enhances subsequent wheat yield and maintains soil carbon storage. Agron. Sustain. Dev. 43, 64. doi: 10.1007/s13593-023-00918-4

[B24] Lu R. K. (2000). Analytical methods for soil and agrochemistry (Beijing: China Agricultural Science and technology Press).

[B25] Mahmoud E. El-Beshbeshy T. El-Kader N. A. El Shal R. Khalafallah N. (2019). Impacts of biochar application on soil fertility, plant nutrients uptake and maize (Zea mays L.) yield in saline sodic soil. Arabian J. Geosciences 12, 1–9. doi: 10.1007/s12517-019-4937-4

[B26] Marx M. C. Wood M. Jarvis S. C. (2001). A microplate fluorimetric assay for the study of enzyme diversity in soils. Soil Biol. Biochem. 33, 1633–1640. doi: 10.1016/S0038-0717(01)00079-7

[B27] Meena M. Joshi P. Narjary B. Sheoran P. Jat H. Chinchmalatpure A.. (2016). Effects of municipal solid waste compost, rice-straw compost and mineral fertilizers on biological and chemical properties of a saline soil and yields in a mustard-pearl millet cropping system. Soil Res. 54, 958–969. doi: 10.1071/SR15342

[B28] Mustafa G. Akhtar M. S. Abdullah R. (2019). Salt stress, microbes, and plant interactions: Causes and solution (Singapore: Springer), 1–19.

[B29] Paz-Ferreiro J. Liu Z. Rong Q. Zhou W. Liang G. (2017). Effects of inorganic and organic amendment on soil chemical properties, enzyme activities, microbial community and soil quality in yellow clayey soil. PloS One 12, e0172767. doi: 10.1371/journal.pone.0172767 28263999 PMC5338777

[B30] Qadir M. Tubeileh A. Akhtar J. Larbi A. Minhas P. Khan M. (2008). Productivity enhancement of salt-affected environments through crop diversification. Land Degradation Dev. 19, 429–453. doi: 10.1002/ldr.v19:4

[B31] Sahab S. Suhani I. Srivastava V. Chauhan P. S. Singh R. P. Prasad V. (2021). Potential risk assessment of soil salinity to agroecosystem sustainability: Current status and management strategies. Sci. Total Environ. 764, 144164. doi: 10.1016/j.scitotenv.2020.144164 33385648

[B32] Shrivastava P. Kumar R. (2015). Soil salinity: A serious environmental issue and plant growth promoting bacteria as one of the tools for its alleviation. Saudi J. Biol. Sci. 22, 123–131. doi: 10.1016/j.sjbs.2014.12.001 25737642 PMC4336437

[B33] Singh B. P. Hatton B. J. Balwant S. Cowie A. L. Kathuria A. (2010). Influence of biochars on nitrous oxide emission and nitrogen leaching from two contrasting soils. J. Environ. Qual. 39, 1224–1235. doi: 10.2134/jeq2009.0138 20830910

[B34] Singh K. Trivedi P. Singh G. Singh B. Patra D. D. (2016). Effect of different leaf litters on carbon, nitrogen and microbial activities of sodic soils. Land Degradation Dev. 27, 1215–1226. doi: 10.1002/ldr.v27.4

[B35] Song D. Tang J. Xi X. Zhang S. Liang G. Zhou W.. (2018). Responses of soil nutrients and microbial activities to additions of maize straw biochar and chemical fertilization in a calcareous soil. Eur. J. Soil Biol. 84, 1–10. doi: 10.1016/j.ejsobi.2017.11.003

[B36] Song J. J. Xu X. Y. Bai J. Z. Yu Q. Cheng B. H. Feng Y. Z.. (2022). Effects of straw return with fertilizer on soil nutrients and winter wheat yield. Environ. Sci. 43, 4839–4847. doi: 10.13227/j.hjkx.202112043 36096624

[B37] Song J. S. Zhang H. Y. Chang F. D. Yu R. Wang J. Chen A. P.. (2024). Subsurface organic amendment of a saline soil increases ecosystem multifunctionality and sunflower yield. Sci. Total Environ. 917, 170276. doi: 10.1016/j.scitotenv.2024.170276 38262534

[B38] Song J. Zhang H. Peixoto L. Chang F. Yu R. Wang X.. (2023). Burying straw interlayers decreases CO2 emissions in deep saline soil. Sustain. Production Consumption 43, 194–203. doi: 10.1016/j.spc.2023.10.022

[B39] Stark S. Männistö M. K. Eskelinen A. (2014). Nutrient availability and pH jointly constrain microbial extracellular enzyme activities in nutrient-poor tundra soils. Plant Soil 383, 373–385. doi: 10.1007/s11104-014-2181-y

[B40] Tian P. Lian H. Wang Z. Jiang Y. Li C. Sui P.. (2020). Effects of deep and shallow tillage with straw incorporation on soil organic carbon, total nitrogen and enzyme activities in northeast China. Sustainability 12, 8679. doi: 10.3390/su12208679

[B41] Turmel M. S. Speratti A. Baudron F. Verhulst N. Govaerts B. (2015). Crop residue management and soil health: a systems analysis. Agric. Syst. 134, 6–16. doi: 10.1016/j.agsy.2014.05.009

[B42] Urraa J. Mijangosa I. Lanzéna A. Lloveras J. Garbisu C. (2018). Effects of corn stover management on soil quality. Eur. J. Soil Biol. 88, 57–64. doi: 10.1016/j.ejsobi.2018.06.005

[B43] Wang S. J. Chen Q. Li Y. Zhuo Y. Q. Xu L. Z. (2017). Research on saline-alkali soil amelioration with FGD gypsum. Resources Conserv. Recycling 121, 82–92. doi: 10.1016/j.resconrec.2016.04.005

[B44] Wang P. Dong J. X. Xia L. L. He J. Kuang S. Xu Y. L.. (2024). Effects of straw carbon types on fungal community characteristics of soil aggregates. Acta Pedologica Sin. 61, 1–18. doi: 10.11766/trxb202309250398

[B45] Wang E. Z. Lin X. L. Tian L. Wang X. G. Ji L. Jin F.. (2021). Effects of short-term rice straw return on the soil microbial community. Agriculture 11, 561. doi: 10.3390/agriculture11060561

[B46] Wittwer R. A. Bender S. F. Hartman K. Hydbom S. Lima R. A. A. Loaiza V.. (2021). Organic and conservation agriculture promote ecosystem multifunctionality. Sci. Adv. 7, 34. doi: 10.1126/sciadv.abg6995 PMC8378818 34417179

[B47] Wu W. Chai H. X. Gao P. L. Gao P. H. Zhang X. Li M. Z.. (2024). Effects of brackish water irrigation and biochar applicationon fertility, enzyme activity, and winter wheat yield in coastalsaline-alkali soils. Land Degradation Dev. 35, 2437–2449. doi: 10.1002/ldr.v35.7

[B48] Xiao D. He X. Wang G. Xu X. Hu Y. Chen X.. (2022). Network analysis reveals bacterial and fungal keystone taxa involved in straw and soil organic matter mineralization. Appl. Soil Ecol. 173, 0929–1393. doi: 10.1016/j.apsoil.2022.104395

[B49] Xie W. J. Chen Q. F. Wu L. F. Yang H. J. Xu J. K. Zhang Y. P. (2020). Coastal saline soil aggregate formation and salt distribution are affected by straw and nitrogen application: a 4-year field study. Soil Tillage Res. 198, 104535. doi: 10.1016/j.still.2019.104535

[B50] Xu C. Han X. Zhuge Y. Xiao G. Ni B. Xu X.. (2020). Crop straw incorporation alleviates overall fertilizer-n losses and mitigates N_2_O emissions per unit applied n from intensively farmed soils: an in *situ* ^15^N tracing study. Sci. Total Environ. 764, 142884. doi: 10.1016/j.scitotenv.2020.142884 33757238

[B51] Xu Y. Liu K. Han Y. Jiang H. Yao S. Zhang B. (2019). A soil texture manipulation doubled the priming effect following crop straw addition as estimated by two models. Soil Tillage Res. 186, 11–22. doi: 10.1016/j.still.2018.09.011

[B52] Xue Y. F. Tian J. Quine T. A. Powlson D. Xing K. X. Yang L. Y. (2020). The persistence of bacterial diversity and ecosystem multifunctionality along a disturbance intensity gradient in karst soil. Sci. Total Environ. 748, 142381. doi: 10.1016/j.scitotenv.2020.142381 33113676

[B53] Zhang H. Y. Pang H. C. Zhao Y. G. Lu C. Liu N. Zhang X. L.. (2020). Water and salt exchange flux and mechanism in a dry saline soil amended with buried straw of varying thicknesses. Geoderma 365, 114213. doi: 10.1016/j.geoderma.2020.114213

[B54] Zhao W. Zhou Q. Tian Z. Z. Cui Y. T. Liang Y. Wang H. Y. (2020). Apply biochar to ameliorate soda saline-alkali land, improve soil function and increase corn nutrient availability in the Songnen Plain. Sci. Total Environ. 722, 137428. doi: 10.1016/j.scitotenv.2020.137428 32197168

[B55] Zhou J. Liu C. Shi L. Zamanian K. (2024). Rhizosphere influence on microbial functions: consequence for temperature sensitivity of soil organic matter decomposition at early stage of plant growth. Plant Soil 494, 95–109. doi: 10.1007/s11104-023-06258-2

[B56] Zhou J. Sun T. Shi L. Kurganova I. de Gerenyu V. L. Kalinina O.. (2023). Organic carbon accumulation and microbial activities in arable soils after abandonment: A chronosequence study. Geoderma 435, 116496. doi: 10.1016/j.geoderma.2023.116496

[B57] Zhou Y. Ma H. B. Xie Y. Z. Jia X. Su T. Li J.. (2020). Assessment of soil quality indexes for different land use types in typical steppe in the loess hilly area, China. Ecolog. Indicat. 118, 106743.

