# Supplementary material for: Straw return was more beneficial to improving saline soil quality and crop productivity than biochar in the short term
Source: Front Plant Sci. 2025 Jan 20;15:1517917. doi: 10.3389/fpls.2024.1517917 (PMC11788349; doi:10.3389/fpls.2024.1517917)
Supplement: Supplementary file 1 [file DataSheet1.docx]

**Fig.S1**

**
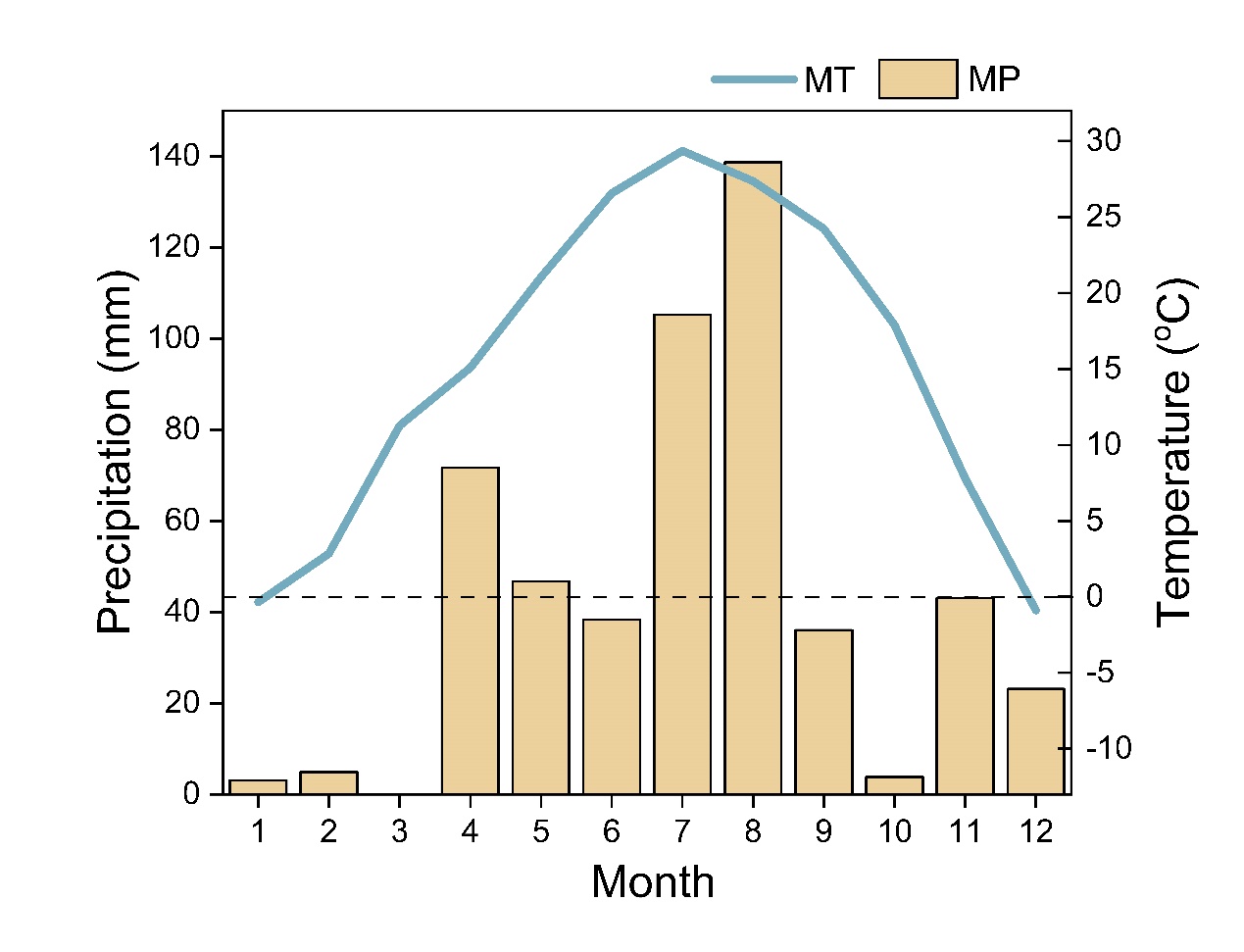
**

**Fig. S1.** Monthly precipitation (MP) and mean air temperature (MAT) in 2023. The dotted line indicated 0 ^o^C.


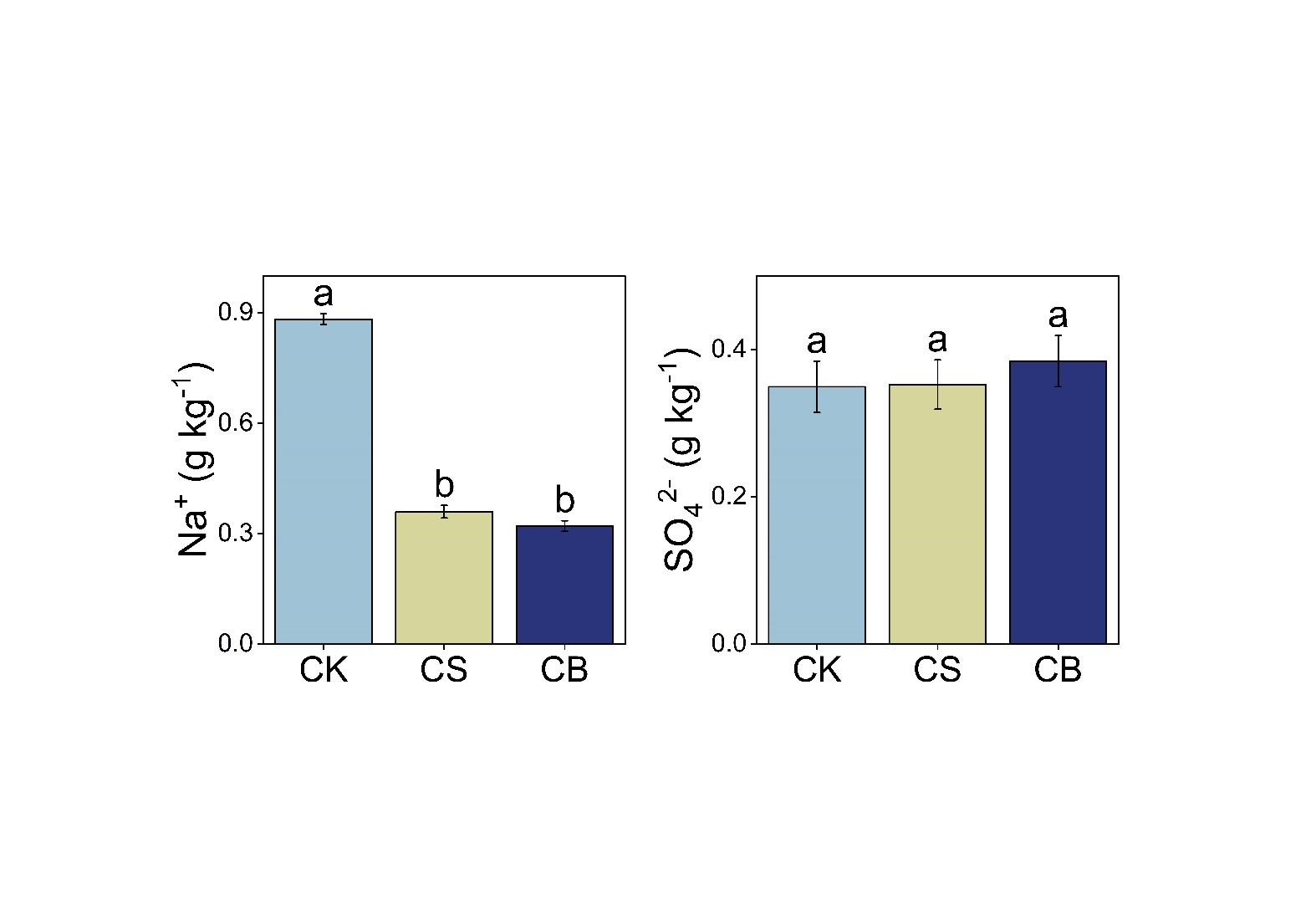


**Fig.S2** Soil solublle Na^+^ and SO_4_^2-^ contents at 0-20 cm as affected by organic ameliorants. organic ameliorants were: CK, no organic ameliorant; CS, corn straw return; CB, corn straw biochar return. Bars were SE and letters were LSD at *p* < 0.05 (n=3).


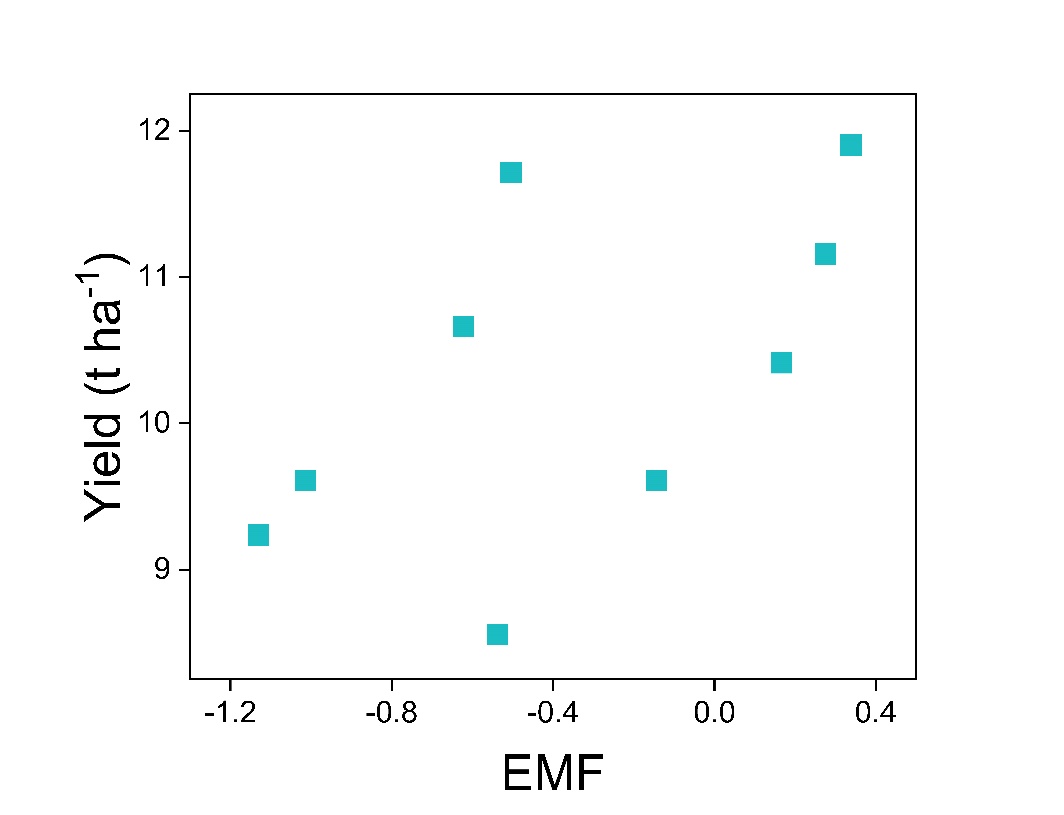


**Fig S2**. The relationship between soil ecosystem multifunctionality (EMF) and yield at 0-20 cm as affected by organic ameliorants.

**Table S1**

**Table S1** Nutrient contents of different ameliorants.

|  | Total N | Total P | Total K | SOC |
| --- | --- | --- | --- | --- |
|  | (g kg^-1^) | (g kg^-1^) | (g kg^-1^) | (g kg^-1^) |
| Straw | 9.12 | 2.82 | 18.55 | 389.10 |
| Straw biochar | 12.78 | 4.36 | 43.18 | 719.35 |

Notes: N, nitrogen; P, phosphorus; K, potassium; SOC, soil organic carbon.

**Table S2**

**Table S2** Testing methods used for soil quality indicators.

| Indicators | Methods |
| --- | --- |
| Salt (g kg^-1^) | Residue drying - quality method. Briefly, 25 mL of soil leachate was placed in a weighed evaporating dish. Evaporate it in a water bath and add 150 g L^-1^H_2_O_2_ until the residue becomes white. Then, the evaporating dish dried in a constant temperature drying oven at 105℃ for 2 h. After weighing, dry it until it reaches a constant weight. Calculate the salt content based on the difference in mass between the two evaporation dishes before and after. (Lu, 2000) |
| Soluble ions (g kg^-1^) | The soluble salt ions in soil were first analyzed by water extract, then Na^+^ and SO_4_^2-^ were determined by flame photometer and EDTA titration, respectively (Bao, 2010). |
| Total N (g kg^-1^) | Kjeldahl nitrogen determination method. Briefly, 0.2 g of air-dried soil sample which sieved through a 0.149 mm sieve was put into a Kjeldahl flask and 5 mL of concentrated sulfuric acid was added. Then, place it in a digestion furnace at a temperature of 375 ℃ and simmer for 1 h until the mixture in the digestion furnace turns a clear light blue color. Cool and clear, take the supernatant, dilute it, adjust the pH value. The total nitrogen content in the solution was measured with Kjeltec nitrogen analyzer (FOSS/Kjeltec 8400, Denmark), and then converted it into soil total N content. (Bao, 2010) |
| SOC (g kg^-1^) | Dry burning method. After removing inorganic carbon from the air-dried soil sample sieved with 100 mesh sieve, 0.2000 g was taken and placed in a sample boat and measured using a TOC analyzer (multi N/C3100, Germany). |
| Available N (mg kg^-1^) | Alkaline hydrolysis diffusion method. Briefly, 2 g of air-dried soil sample was placed in the outer chamber of a diffusion dish. And 10 mL of 1M NaOH solution was added to the inner chamber of the diffusion dish, quickly sealed, and subjected to alkaline hydrolysis diffusion reaction at 40 ℃ for 24 h. Ammonia in the absorption solution was titrated with 0.01M HCI solution, and the soil available N content was calculated based on the amount of hydrochloric acid consumed. (Lu, 2000) |
| Available P (mg kg^-1^) | Sodium bicarbonate extraction molybdenum antimony colorimetric method. Simply put, weigh 2.5 g of air dried soil sample that has passed through a 2 mm sieve into a 150 mL conical flask, add 50mL of 0.5M NaHCO_3_ solution, shake for 30 minutes (180 r min^-1^), filter through filter paper, collect the filtrate, take 5mL of filtrate, add ammonium molybdate ascorbic acid mixed reagent, and after reaction, compare the color at 700 nm wavelength. A spectrophotometer was used to measure the absorbance and calculate the effective phosphorus content in the soil. (Lu, 2000) |
| Available K (mg kg^-1^) | Ammonium acetate extraction flame photometer method. Briefly, 5 g of air dried soil sample had been passed through a 2 mm sieve and placed it in a 250 mL conical flask. Add 50 mL of 1M NH_4_OAc solution and shake for 30 mins (120 r min^-1^). Filter the mixture, collect the filtrate, and use a flame photometer to measure the potassium content in the filtrate. (Lu, 2000) |
| BG (nmol g^−1^ h^−1^) | Fluorescence methods. Briefly, 1 g fresh of soil sample was weighed, and 50 ml distilled water was added and then shaken in shaker for 30 mins. After agitation, soil suspension (50 μL), buffer (50 μL), and the corresponding substrate (100 μL) at a concentration of 400 μmol g^-1^ were added to the 96-well microplates. Fluorescence measurements (360 and 450 nm) were conducted after 0.5, 1, and 2 hr using a fluorescence microplate reader (Thermo Fisher Scientific, USA) (Marx et al., 2001) |
|  |  |
| CE (nmol g^−1^ h^−1^) |  |
| NAG (nmol g^−1^ h^−1^) |  |
| LAP (nmol g^−1^ h^−1^) |  |

Notes: N, nitrogen; P, phosphorus; K, potassium; SOC, soil organic carbon; BG, β-1, 4-glucosidase; CE, cellobiosidase; NAG, β-1,4-N-Acetyl-glucosaminidase; LAP, Leucine aminopeptidase.

**References**

Bao, S.D. (2010). Soil Agrochemical Analysis. Beijing: China Agricultural Press 495.

Marx, M.C., Wood, M., Jarvis, S.C. (2001). A microplate fluorimetric assay for the study of enzyme diversity in soils. *Soil Biology Biochemistry* 33, 1633-1640.

Lu, R.K. (2000). Analytical methods for soil and agrochemistry. Beijing: China Agricultural Science and technology Press.
